# A predictive model of response to metoprolol in children and adolescents with postural tachycardia syndrome

**DOI:** 10.1007/s12519-022-00677-4

**Published:** 2023-02-13

**Authors:** Bo-Wen Xu, Qing-You Zhang, Xue-Ying Li, Chao-Shu Tang, Jun-Bao Du, Xue-Qin Liu, Hong-Fang Jin

**Affiliations:** 1 Department of Pediatrics, Peking University First Hospital, No. 1, Xi’an-Men Street, West District, Beijing, 100034 China; 2 Department of Medical Statistics, Peking University First Hospital, Beijing, China; 3 Key Lab of Molecular Cardiovascular Sciences, Ministry of Education, Beijing, China; 4 Department of Physiology and Pathophysiology, Peking University Health Science Centre, Beijing, China

**Keywords:** Children, Electrocardiography, Metoprolol, Nomogram, Predictor, Postural tachycardia syndrome

## Abstract

**Background:**

The present work was designed to explore whether electrocardiogram (ECG) index-based models could predict the effectiveness of metoprolol therapy in pediatric patients with postural tachycardia syndrome (POTS).

**Methods:**

This study consisted of a training set and an external validation set. Children and adolescents with POTS who were given metoprolol treatment were enrolled, and after follow-up, they were grouped into non-responders and responders depending on the efficacy of metoprolol. The difference in pre-treatment baseline ECG indicators was analyzed between the two groups in the training set. Binary logistic regression analysis was further conducted on the association between significantly different baseline variables and therapeutic efficacy. Nomogram models were established to predict therapeutic response to metoprolol. The receiver-operating characteristic curve (ROC), calibration, and internal validation were used to evaluate the prediction model. The predictive ability of the model was validated in the external validation set.

**Results:**

Of the 95 enrolled patients, 65 responded to metoprolol treatment, and 30 failed to respond. In the responders, the maximum value of the P wave after correction (Pcmax), P wave dispersion (Pd), Pd after correction (Pcd), QT interval dispersion (QTd), QTd after correction (QTcd), maximum T-peak-to-T-end interval (Tpemax), and T-peak-to-T-end interval dispersion (Tped) were prolonged (all *P* < 0.01), and the P wave amplitude was increased (*P* < 0.05) compared with those of the non-responders. In contrast, the minimum value of the P wave duration after correction (Pcmin), the minimum value of the QT interval after correction (QTcmin), and the minimum T-peak-to-T-end interval (Tpemin) in the responders were shorter (*P* < 0.01, < 0.01 and < 0.01, respectively) than those in the non-responders. The above indicators were screened based on the clinical significance and multicollinearity analysis to construct a binary logistic regression. As a result, pre-treatment Pcmax, QTcmin, and Tped were identified as significantly associated factors that could be combined to provide an accurate prediction of the therapeutic response to metoprolol among the study subjects, yielding good discrimination [area under curve (AUC) = 0.970, 95% confidence interval (CI) 0.942–0.998] with a predictive sensitivity of 93.8%, specificity of 90.0%, good calibration, and corrected C-index of 0.961. In addition, the calibration curve and standard curve had a good fit. The accuracy of internal validation with bootstrap repeated sampling was 0.902. In contrast, the kappa value was 0.769, indicating satisfactory agreement between the predictive model and the results from the actual observations. In the external validation set, the AUC for the prediction model was 0.895, and the sensitivity and specificity were 90.9% and 95.0%, respectively.

**Conclusions:**

A high-precision predictive model was successfully developed and externally validated. It had an excellent predictive value of the therapeutic effect of metoprolol on POTS among children and adolescents.

**Supplementary Information:**

The online version contains supplementary material available at 10.1007/s12519-022-00677-4.

## Introduction

Postural tachycardia syndrome (POTS) is a common syndrome that significantly affects the quality of life among children and adolescents [1, 2]. POTS shows a 6.8% prevalence in Chinese children [3]. It is characterized by chronic orthostatic intolerance with symptoms including palpitations, dizziness, chest tightness, gastrointestinal symptoms, etc. [1, 2]. The pathogenesis of POTS is unclear but mainly involves increased catecholamine contents and sympathetic hyperfunction, hypovolemia, excessive vasodilation, and immune abnormalities [4]. Empirical and nonselective beta-adrenoceptor blocker treatment among all children and adolescents with POTS has limited efficacy. For instance, metoprolol was previously depicted to achieve a low effective rate of only around 50% among POTS in children [5]. Since metoprolol blocks β-adrenoceptors within cardiovascular tissues to reduce excessive sympathetic nerve activity in POTS, it would be only effective for POTS patients with high plasma catecholamine levels and sympathetic activity as the main pathogenesis [6]. Therefore, if we can effectively predict POTS patients with elevated pre-treatment plasma catecholamine levels or increased sympathetic activity, individualized treatment with beta-adrenoceptor blockers would have a favorable therapeutic efficacy in such instances of POTS.

To predict whether high sympathetic activity exists among pediatric POTS before treatment, the investigators showed that the pre-treatment plasma norepinephrine levels [7], copeptin levels [8], C-type natriuretic peptide levels [9], baseline values of pre-treatment 24-hour heart rate variability [10], QTcd [11], and heart rate variation [12] during head-up tilt testing (HUTT) had a specific predictive value for the effectiveness of metoprolol in treating POTS. However, detecting plasma norepinephrine [7], plasma copeptin [8], and C-type natriuretic peptide [9] is complex and requires blood collection through an invasive procedure. The 24-hour heart rate variability examination [10] is time-consuming, and HUTT [12] is expensive, and requires specialized equipment. QTcd [11], as a single ECG index, has low predictive specificity and sensitivity in predicting the therapeutic response to beta-adrenoceptor blockers. Therefore, it is essential to identify valuable indicators or models that are easy to use and inexpensive with high sensitivities and specificities in predicting the effectiveness of metoprolol among pediatric POTS. Electrocardiographic waveforms can reflect the effects of sympathetic and vagal interactions on the heart. Sympathetic excitation is associated with elevated heart rate, prolonged P wave duration, enhanced P wave amplitude, shortened QT interval, low flattened or inverted T wave, extended T-peak-to-T-end interval (Tpe interval), and high dispersion in ECG [13–16].

Therefore, the study was conducted to comprehensively analyze the pre-treatment ECG indices associated with sympathetic nervous function in POTS children to determine if the integrated indices derived from ECG would successfully predict the effectiveness of metoprolol on pediatric POTS to improve the effective rate of individualized therapy with metoprolol among pediatric patients with POTS.

## Methods

### Source of data and participants

**Fig. 1:**
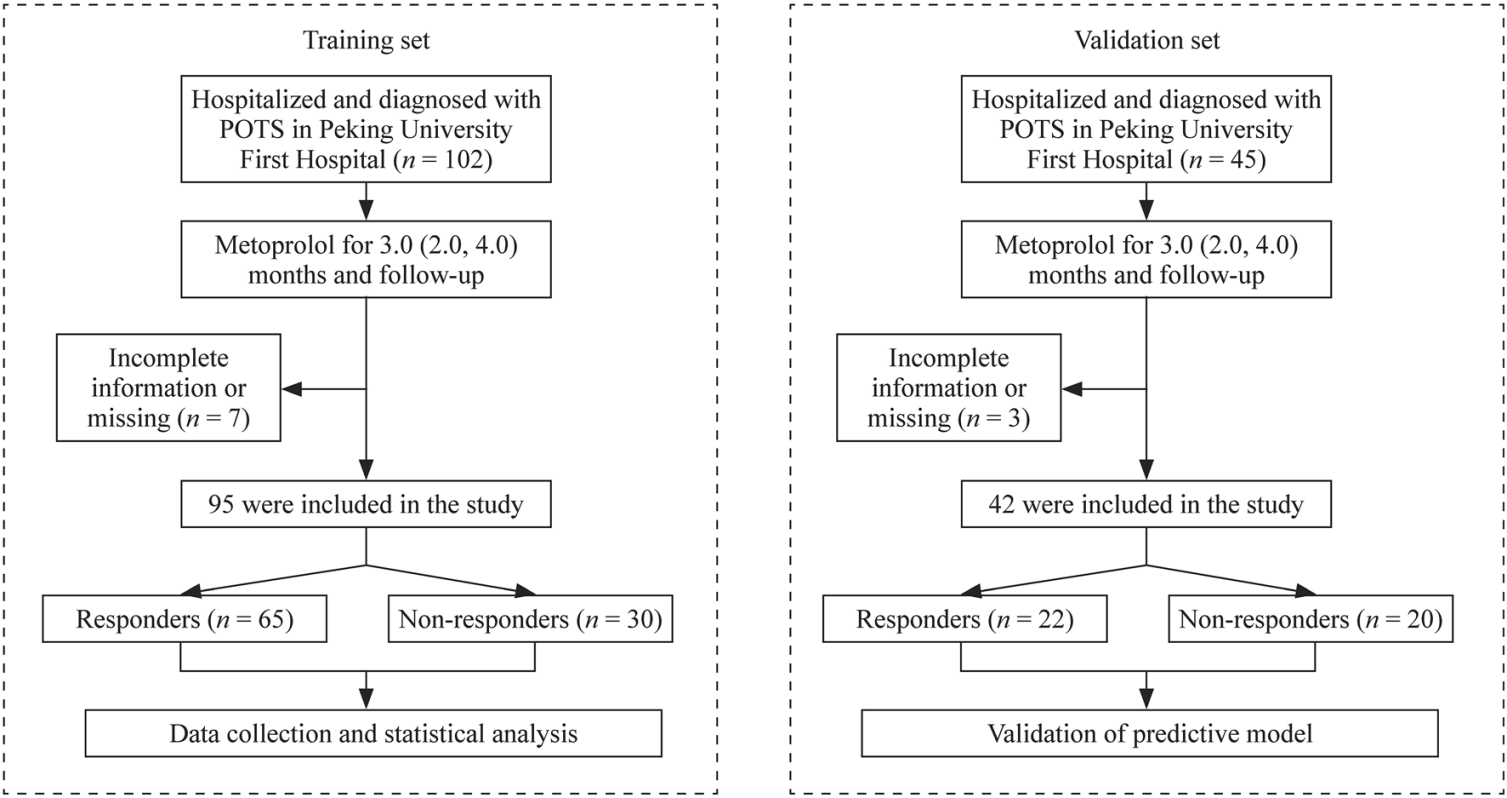
Flowchart of the inclusion of participants in the study. *POTS* postural tachycardia syndrome

We screened 147 patients with POTS, of whom 137 (93.2%) were eligible. The study consisted of a training set and an external validation set. In the training set, a retrospective analysis of 102 POTS among children and adolescents receiving metoprolol treatment was conducted, and the patients were followed up at the Department of Pediatrics, Peking University First Hospital, China, between January 2012 and December 2020. Seven children with POTS were excluded from this study because of lost to follow up or inadequate information during the follow-up. The loss to follow-up rate was 6.9%. Therefore, we enrolled 95 POTS children receiving oral metoprolol treatment in the training set. In addition, the present study enrolled another 42 patients who met the inclusion criteria and received metoprolol treatment between January 2021 and June 2022 in the external validation set. Figure 1 shows the flowchart of the patients inclusion.

The diagnostic criteria for POTS included (1) symptoms related to predisposing factors, including the extended standing position or a fast change from supine to upright; (2) symptoms of orthostatic intolerance, including headache, dizziness, blurred vision, fatigue, palpitation, chest tightness, syncope following the upright position, exercise intolerance, and hand tremors; (3) positive standing or HUTT test results, and (4) exclusion of cardiovascular, nervous, and metabolic diseases [17].

Inclusion criteria were:  (1) children and adolescents aged 5–18 years; (2) those diagnosed with POTS in Peking University First Hospital, China; (3) all received a standard assessment, including complete medical history, physical/neurological examination, baseline laboratory assessment, 12-lead ECG, echocardiography, standing test, or HUTT, and (4) the patients receiving the treatment with metoprolol. Exclusion criteria were: (1) cardiac syncope or orthostatic intolerance due to cardiac, neurologic, or metabolic diseases; (2) patients without sinus rhythm on ECG; (3) those without complete medical records or data, and (4) those who were lost to follow-up.

The Ethics Committee of Peking University First Hospital, China approved the investigation. Written informed consent was obtained from the parents/guardians of the participants in this study.

### Data collection

#### Demographic and clinical data

We secured medical records of all the patients through the Medical Recording Management Digital System (Kaihua, Beijing, China), including demographic data [age, sex, and body mass index (BMI)], detailed medical history (previous medical history, history of present illness, family history, or any allergic history), physical examination, hemodynamic parameters [supine heart rate (HR), systolic blood pressure (SBP), and diastolic blood pressure (DBP)], and the results of laboratory investigations (blood biochemistry, echocardiography, 24-hour Holter monitoring, cranial CT, or MRI).

#### Symptom score

The symptom score (SS) was recorded for all the patients. SS was obtained based on the orthostatic intolerance symptom frequency, including syncope, dizziness, chest tightness, headache, palpitations, sweating, gastrointestinal symptoms, blurred vision or amaurosis, hand tremors, and difficulties in attention centralization. Symptom frequency was scored as follows [18, 19]: 0 point represented no appearance of orthostatic intolerance symptoms; 1 point indicated symptoms once a month; 2 points represented symptoms 2–4 times a month, 3 points represented symptoms 2–7 times a week, and 4 points indicated symptom frequency greater than once per day. The symptom scores were evaluated to obtain the overall SS for each patient.

#### Standing and basic head-up tilt tests

The procedure for standing test [20]: the patient was in a dimly lit and quiet environment within an appropriate temperature and rested under a supine position for a 10-minute period until the heart rate was stable. Then, the patient was requested to lie upright and stand for a 10-minute period. HR and blood pressure (BP) were dynamically recorded with a monitor (Dash 2000, General Electric Company, NY, USA) throughout this procedure to visualize and record whether the subject showed any discomfort during the test. If the patient could not tolerate the test, it was immediately stopped.

The procedure for basic HUTT [11, 21]: any drug and diets possibly affecting autonomic nervous function, such as tea and coffee, which may influence autonomic nervous function, were discontinued for over five half-lives before the test. The patients were required to fast and abstain for 4 hours before the test. The examination was scheduled from 8:00 to 11:00 am in a quiet environment at appropriate room temperature and dim light. The HUTT was performed using a tilting bed (SHUT-100A, Standard, Jiangsu-HUT-821, Juchi Company, Beijing, China). ECG and heart rate were continuously monitored with a multi-lead ECG monitor (General Electric, NY, USA). Hemodynamic changes were determined with the non-invasive continuous BP monitor Finapres Medical system-FMS (FinometerPRO, FMS, Amsterdam, The Netherlands). The patient was kept in a supine and quiet position for 10–30 minutes, followed by continuous measurement of basic ECG and BP. When the HR, BP, and ECG data were kept stable, the patient was tilted at 60°. The above indicators were continuously recorded until a positive response was achieved or after 45 minutes of the test. Once a positive response occurred, the patient was again placed supine.

#### Electrocardiography

All patients were required to complete the 12-lead ECG by electrocardiography (FX-7402, Fukuda, Japan). ECG was recorded after the patient was stable in the supine position for at least 10 min in the quiet room. Then, quiet and comfortable breathing was encouraged for the patients within this trial. The paper determined the ECG results at 1 mV/cm (25 mm/s), followed by scanner digitization. ECG parameters were determined with Image-Pro Plus version 6.0.0.260 on a high-resolution computer screen at a threefold magnification of the stored digitized ECG data. ECG parameters were selected based on the ECG during sinus rhythm. We selected the horizontal line at the beginning of the Q wave as the isoelectric baseline to measure the amplitude of each waveform of the electrocardiogram, and if the beginning of the Q wave was not clear, we used the TP segment or PR segment for measurement [22–24]. P wave amplitude represents the distance from the isoelectric line position of lead II to the apex of the P wave. The P wave duration indicated the period from the beginning to the end of the P wave. The QT interval represented the duration between the QRS wave beginning and the end of the T wave, while the Tpe interval was the time from the apex of the T wave to its end in ECG. Thus, the T wave apex was the intersection between the highest vertical peak of the upright T wave and the isoelectric line or the lowest vertical valley within the inverted T wave with the isoelectric line. The T wave endpoint became the intersection of the descending branch through the isoelectric line. Pmax, Pmin, QTmax, QTmin, Tpemax, and Tpemin were determined in 12-lead ECG and expressed in milliseconds. Based on the Bazett formula, the corrected Pmax (Pcmax = Pmax/RR^1/2^), Pmin (Pcmin = Pmin/RR^1/2^), QTmin (QTcmin = QTmin/RR^1/2^), and QTmax (QTcmax = QTmax/RR^1/2^) were obtained after correcting the heart rate. Indicators representing dispersion (including Pd, Pcd, QTd, QTcd, and Tped) were obtained by calculating the difference between the maximum and minimum values in 12-lead ECG. Measurements are listed as the mean values of three-to-five consecutive R–R cycles.

Medical history and laboratory results were reviewed and documented by a dedicated investigator for all the patients. Another investigator independently proofread the records.

### Treatment, follow-up, and outcome

In this study, all the children and adolescents diagnosed with POTS were provided health education to allow them and their families to understand POTS fully, avoid predisposing factors, enhance water and salt intake, and strengthen autonomic nervous function exercises (limb compression movements and orthostatic training). Metoprolol was given to children and adolescents who still showed symptoms of orthostatic intolerance after receiving standardized health education and physical treatment. Metoprolol was initially provided at 0.5 mg/kg twice a day, and the course of treatment was 3 (2, 4) months according to the tolerance of the patient (maximum, 2 mg/kg/day) [25].

The POTS patients received a follow-up of 3 months after metoprolol therapy and were documented by trained personnel. In addition, follow-up was performed by hospital visits and telephone follow-up. Medication compliance, frequency of orthostatic intolerance symptoms, and side effects were carefully monitored during the follow-up period.

The cases were grouped into responder and non-responder groups based on the effectiveness of metoprolol treatment at follow-up. Pre-treatment SS was evaluated after the cases were initially diagnosed as POTS. SS after treatment was determined after the follow-up. A decrease of 50% or more in SS after treatment relative to SS before treatment was considered effective and ineffective if the decrease was less than 50% [26].

### Statistical analysis

#### Univariate regression

The normally distributed measurement data were represented as mean ± SD. If the data were not within a normal distribution, they were expressed by the median and interquartile range (IQR). For continuous variables, normality was tested using the Kolmogorov–Smirnov test. Intergroup differences were compared with Student’s *t* test or the Mann–Whitney *U* test based on the normal distribution of continuous variables. For categorical variables, the difference was tested using the *χ*^2^ test, and categorical variables were expressed as values and percentages (%). *P* < 0.05 (two-sided) was considered statistically significant.

#### Establishment of the predictive model

Factors that showed statistical significance during univariable regression were screened according to clinical significance and multicollinearity analysis. The significant variables were incorporated into a binary logistic regression, where two-sided *P* values < 0.05 were used to identify independent factors. The combination of factors most accurately associated with metoprolol efficacy was determined by backward stepwise analysis. Their odds ratios (ORs) and 95% confidence intervals (CIs) were calculated. A nomogram was created based on the binary logistic regression model.

#### Evaluation and validation

The area under the curve (AUC) was used to evaluate the nomogram discrimination in the study, providing the model accuracy. The calibration curve and Hosmer–Lemeshow goodness-of-fit test were applied to assess the goodness-of-fit between the predicted model and the observed data. Then, the corrected C-index was calculated, which was the consistency of the model. Bootstrap repeated sampling (1000 bootstrap resamplings) was performed for internal validation to reduce the over-fitting bias of the nomogram model and determine accuracy and kappa values. The model was used to assess the score of children with POTS in the validation set to predict the efficacy of metoprolol, and ROC curves and calibration curves were plotted to verify the accuracy and reproducibility of the prediction model. Statistical analysis was conducted with SPSS 24.0 (IBM, New York, USA) and R software (version: 4.2.0, Fig. http://www.R-project.org).

## Results

### Clinical features and hemodynamic parameters for subjects in the training set

In this study, 95 children (44 boys and 51 girls) with POTS in the training set were analyzed. The responder group consisted of 33 (50.8%) boys and 32 (49.2%) girls, and the non-responder group consisted of 11 boys (36.7%) and 19 girls (63.3%). Data of sex, age, BMI, supine HR, SBP, DBP, and pre-treatment SS between the two groups did not significantly differ (*P* > 0.05). Table 1 represents the patient clinical features.

**Table 1:** Demographic and hemodynamic parameters between responders and non-responders to metoprolol in children with POTS in the training set

Items	Responders	Non-responders	*t*/*Z*/*χ*^2^ value	*P* value
Number *n* (%)	65 (68.4)	30 (31.6)	NA	NA
Sex (M/F)	33/32	11/19	1.642	0.200
Age (y)	12.0 (10.0, 13.0)	13.0 (11.0, 13.0)	−0.919	0.358
BMI (kg/cm^2^)	20.2 (16.9, 23.8)	19.0 (16.9, 20.9)	−0.993	0.321
Supine HR (bpm)	77.0 (70.5, 87.5)	76.5 (71.0, 87.3)	−0.357	0.721
SBP (mmHg)	114.8 ± 13.1	113.8 ± 8.5	0.376	0.708
DBP (mmHg)	70.0 (61.5, 75.5)	68.0 (65.8, 73.0)	−0.204	0.838
Pre-treatment SS (points)	7.0 (4.0, 12.0)	7.5 (4.0, 10.0)	−0.269	0.788

*POTS* postural tachycardia syndrome, *M/F* male/female, *BMI* body mass index, *HR* heart rate, *bpm* beats per minutes, *SBP* systolic blood pressure, *DBP* diastolic blood pressure, *SS* symptom score, *NA* not available

### Model development in the training set

In univariable analysis, baseline Pcmax, Pd, Pcd, QTd, QTcd, Tpemax, and Tped in responders were elevated compared with those in non-responders (all *P* < 0.01); P wave amplitude in responders was enhanced compared with that in non-responders (*P* < 0.05), and Pcmin, QTcmin, and Tpemin in responders were reduced compared with those in non-responders (all *P* < 0.01, Table 2).

**Table 2:** Comparison of baseline ECG parameters between responders and non-responders to metoprolol in children with POTS in the training set

Items	Responders	Non-responders	*t*/*Z* value	*P* value
P wave amplitude (mV)	0.119 (0.100, 0.132)	0.109 (0.081, 0.133)	−1.974	0.048
Pcmax (ms)	117.2 ± 13.5	104.5 ± 13.1	4.292	< 0.01
Pcmin (ms)	61.6 ± 10.8	70.9 ± 8.7	−4.133	< 0.01
Pd (ms)	48.9 ± 10.6	29.5 ± 7.9	8.965	< 0.01
Pcd (ms)	55.6 ± 11.6	33.6 ± 8.9	9.157	< 0.01
QTcmax (ms)	452.1 ± 28.9	441.5 ± 23.4	1.768	0.080
QTcmin (ms)	369.7 ± 30.3	393.0 ± 23.2	−3.736	< 0.01
QTd (ms)	70.8 (52.2, 89.1)	38.6 (32.2, 46.1)	−5.501	< 0.01
QTcd (ms)	78.1 (62.5, 102.3)	43.3 (37.6, 57.3)	−5.476	< 0.01
Tpemax (ms)	109.7 (100.0, 119.3)	96.7 (88.4, 104.2)	−4.328	< 0.01
Tpemin (ms)	50.5 ± 10.0	59.9 ± 9.3	−4.385	< 0.01
Tped (ms)	56.7 (51.2, 70.1)	36.8 (31.4, 41.3)	−6.870	< 0.01

*ECG* electrocardiogram, *POTS* postural tachycardia syndrome, *Pcmax* the maximum value of P wave duration in 12 leads of ECG after correction, *Pcmin* the minimum value of P wave duration in 12 leads of ECG after correction, *Pd* P wave duration dispersion, *Pcd* Pd after correction, *QTcmax* the maximum value of QT interval in 12 leads of ECG after correction, *QTcmin* the minimum value of QT interval in 12 leads of ECG after correction, *QTd* QT interval dispersion *QTcd* QTd after correction, *Tpemax* the maximum value of T-peak-to-T-end interval in 12 leads of ECG, *Tpemin* the minimum value of T-peak-to-T-end interval in 12 leads of ECG, *Tped* T-peak-to-T-end dispersion

Multicollinearity analysis was performed between the above parameters, and the parameters Pcmax, QTcmin, and Tped which were not correlated with each other were used for the subsequent analysis (Supplemental Table 1). The binary logistic regression indicated that pre-treatment Pcmax, QTcmin, and Tped were the variables associated with the effectiveness of metoprolol therapy in POTS patients (Supplemental Table 2).

The AUCs of baseline Pcmax, QTcmin, and Tped in predicting the response to metoprolol were 0.777 (95% CI 0.671–0.883), 0.738 (95% CI 0.633–0.843), and 0.940 (95% CI 0.887–0.993), respectively. In cases of the greatest Youden index, pre-treatment baseline cutoffs for Pcmax, QTcmin, and Tped were 109 ms, 382.5 ms, and 45.6 ms, respectively, yielding sensitivities of 76.9%, 70.8%, and 92.3% and specificities of 76.7%, 76.7%, and 90.0%, respectively, for predicting the treatment efficacy (Table 3).

**Table 3:** The cut-off value of predictor variables predicting metoprolol efficacy in children with POTS in the training set

Variables	AUC	95% CI	*P* value	Cut-off value	Sensitivity	Specificity
Pcmax	0.777	0.671–0.883	< 0.01	≥ 109 ms	76.9%	76.7%
QTcmin	0.738	0.633–0.843	< 0.01	≤ 382.5 ms	70.8%	76.7%
Tped	0.940	0.887–0.993	< 0.01	≥ 45.6 ms	92.3%	90.0%
Combined Prediction	0.970	0.942–0.998	< 0.01	≥ 0.266	93.8%	90.0%

*POTS* postural tachycardia syndrome, *ECG* electrocardiogram, *Pcmax* the maximum value of P wave duration in 12 leads of ECG after correction, *QTcmin* the minimum value of QT interval in 12 leads of ECG after correction, *Tped* T-peak-to-T-end dispersion, *AUC* area under receiver-operating characteristic curve, *CI* confidence interval

**Fig. 2:**
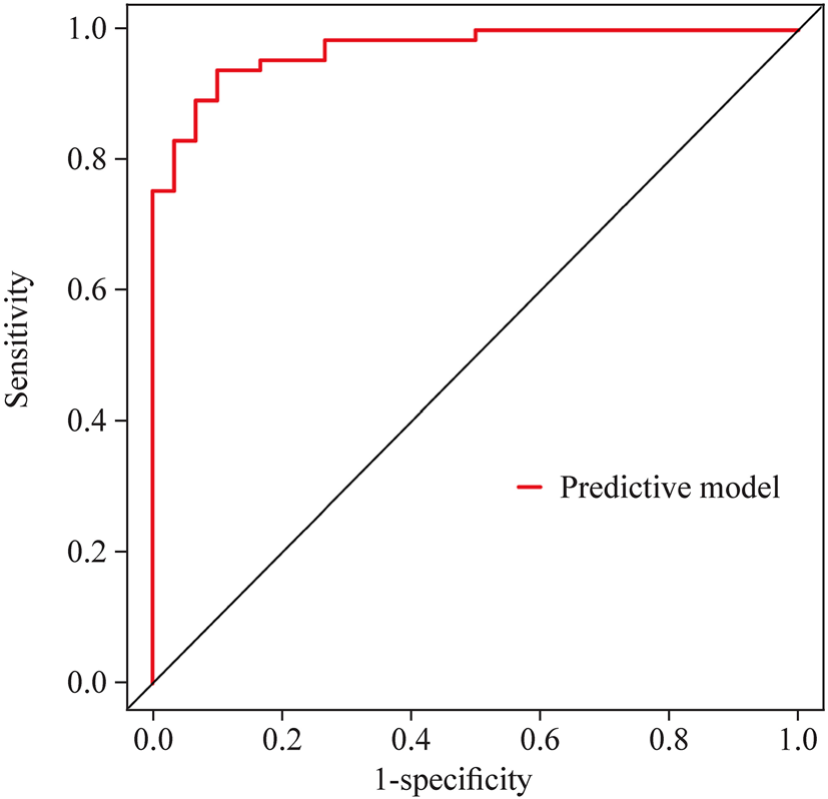
An ROC analysis of the nomogram for evaluating the effectiveness of metoprolol in POTS in children in the training set. The *y*-axis represents the sensitivity to predict the response to metoprolol; the *x*-axis represents the specificity to predict the response to metoprolol. The 45° reference line of the chart indicates that the sensitivity and the specificity are equal to 50%. The area under the curve was 0.970 with a 95% confidence interval of 0.942–0.998. *ROC* receiver-operating characteristic curve, *POTS* postural tachycardia syndrome, *AUC* area under the curve

To further improve the predictive values, these variables were combined to predict the efficacy of metoprolol. A model was constructed based on logistic regression as follows: *Y* = Logit (P) =  − 2.258 + 0.073 × Pcmax − 0.040 × QTcmin + 0.208 × Tped, where P was the probability value to predict the efficacy of metoprolol. Pcmax, QTcmin, and Tped were taken as the actual measured values. The AUC for combined prediction was 0.970 (95% CI 0.942–0.998, Fig. 2). Moreover, the optimum prediction cut-off *Y* = 0.266 (*P* = 0.566) was determined using the Youden index, which generated a predictive sensitivity and specificity of 93.8% and 90.0%, respectively (Table 3).

### Establishment of a nomogram in the training set

**Fig. 3:**
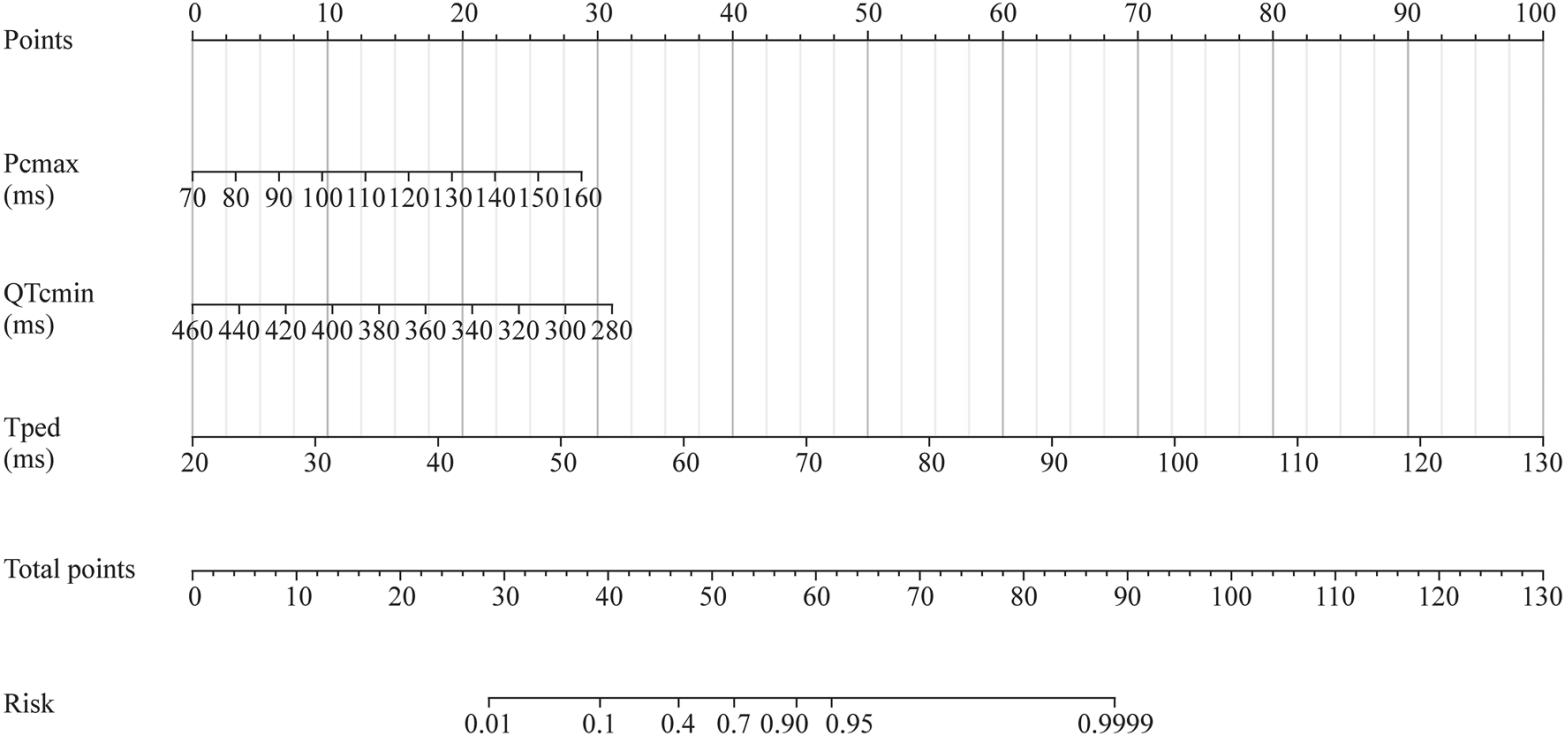
Nomogram for predicting the efficacy of metoprolol in children with POTS in the training set. To estimate the response to metoprolol, the patient score for each axis is marked, a line perpendicular to the point axis is drawn, and the points for all variables are summed. Next, the sum is marked on the total point axis, and a line perpendicular to the probability axis is drawn. *POTS* postural tachycardia syndrome, *Pcmax* the maximum value of P wave duration in 12 leads of electrocardiogram after correction, ms millisecond, *QTcmin* the minimum value of QT interval in 12 leads of electrocardiogram after correction, *Tped* T-peak-to-T-end dispersion

A nomogram model was constructed based on binary logistic regression analysis (Fig. 3). The model revealed that the effective rate of metoprolol treatment increased with the prolongation of Pcmax and Tped and the shortening of QTcmin before treatment.

### Evaluation of the model in the training set

**Fig. 4:**
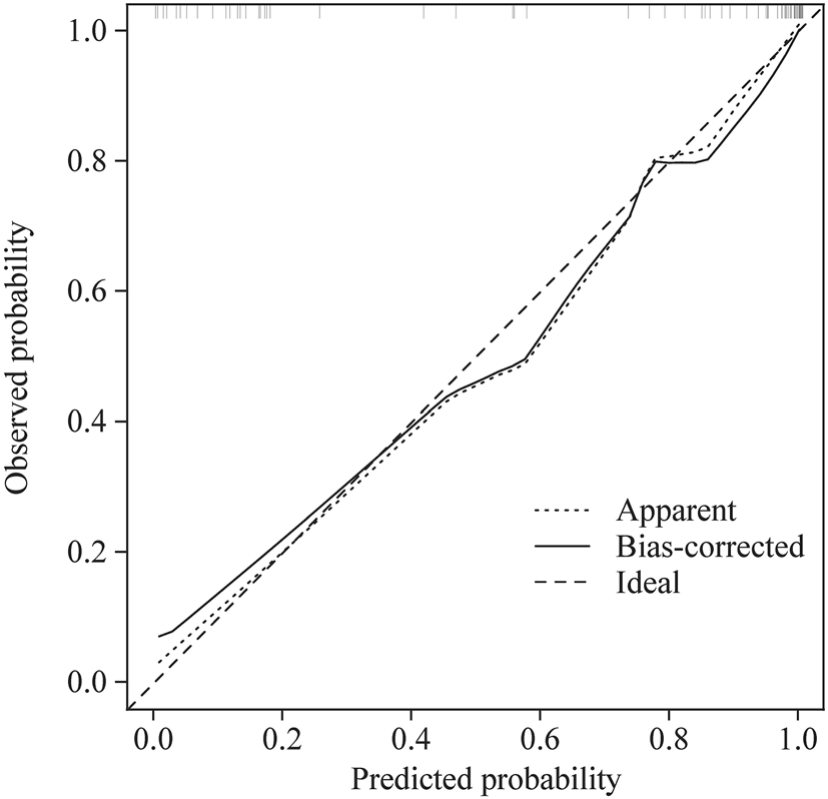
Calibration of the nomogram for predicting the efficacy of metoprolol in children with POTS in the training set. The *x*-axis shows the predicted probability of metoprolol response, and the *y*-axis shows the observed probability of metoprolol response. The ideal line means that the predicted and actual probabilities of the model agree perfectly. The apparent line indicates the actual performance of the prediction model in the training set. The bias-corrected line indicates the performance of the prediction model in the training set after the correction of the over-fitting situation. The calibration curve and standard curve have a good fit. *POTS* postural tachycardia syndrome

The ROC curve developed by the predictive model was the same as described earlier in the HL test, and the *χ*^2^ of the prediction model was 0.9196 (*P* = 0.6314 > 0.05). The corrected C-index was 0.961 after evaluation. The calibration curve of the nomogram was drawn (Fig. 4). The calibration curve and standard curve had a good fit.

### Internal validation and external validation

**Fig. 5:**
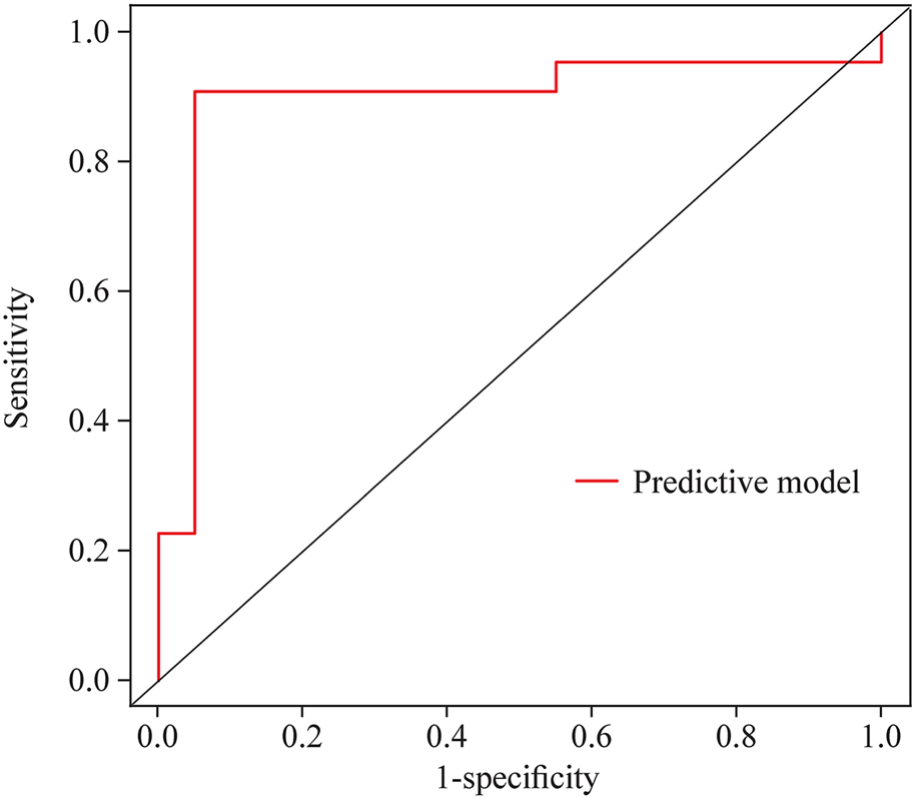
An ROC analysis of the nomogram for evaluating the effectiveness of metoprolol in POTS in children in the external validation set. The *y*-axis represents the sensitivity to predict the response to metoprolol; the *x*-axis represents the specificity to predict the response to metoprolol. The 45° reference line of the chart indicates that the sensitivity and the specificity are equal. The AUC was 0.895, and the sensitivity and specificity were 90.9% and 95%, respectively. *ROC* receiver-operating characteristic curve, *POTS* postural tachycardia syndrome, *AUC* area under the curve

**Fig. 6:**
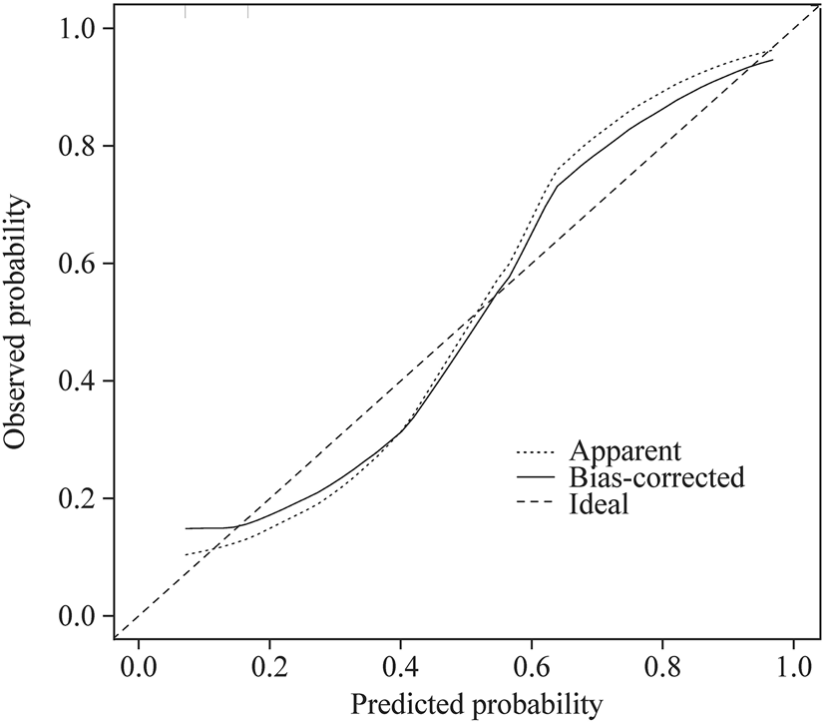
Calibration of the nomogram for predicting the efficacy of metoprolol in children with POTS in the external validation set. The *x*-axis shows the predicted probability of metoprolol response, and the *y*-axis shows the observed probability of metoprolol response. The ideal line means that the predicted and actual probabilities of the model agree perfectly. The apparent line indicates the actual performance of the prediction model in the validation set. The bias-corrected line indicates the performance of the prediction model in the validation set after the correction of the overfitting situation. The calibration curve and standard curve have a good fit. *POTS* postural tachycardia syndrome

Finally, we performed internal validation by bootstrap repeated sampling, having an accuracy of 90.2% and a kappa value of 0.769. In the validation set of children with POTS, the AUC for the prediction model was 0.895, and the sensitivity and specificity were 90.9% and 95.0%, respectively. The ROC curves and calibration curves are shown in Figs. 5 and 6.

## Discussion

In this study, pre-treatment Pcmax, QTcmin, and Tped could be used to predict the effectiveness of metoprolol on pediatric POTS. A combined prediction of the three indices could more accurately predict the efficacy of metoprolol. A nomogram model was developed to predict the response to metoprolol among pediatric patients. Our constructed model exhibited excellent discrimination. The AUC was 0.970 (95% CI 0.942–0.998), yielding a predictive sensitivity and specificity of 93.8% and 90.0%, respectively, at the *Y* = 0.266 (*P* = 0.566) cutoffs. The corrected C-index was 0.961. The results indicated that it was well calibrated, and the internally validated model was not overfitted with good clinical application value. In the external validation, the sensitivity and specificity of the prediction model were 90.9% and 95.0%, respectively, which were similar to the results obtained in the training set, indicating that this predictive model had good practical value in the application.

Pediatric POTS is a chronic syndrome that strongly affects the quality of life of patients. Current studies have reported that pathogenesis, including excessive sympathetic function or plasma catecholamine levels, impaired limb muscle pump function, vascular endothelial dysfunction, or effective circulating hypovolemia, could be involved in developing POTS [27]. Recently, Zhang et al. revealed that baseline norepinephrine was remarkably elevated among some cases with POTS, depicting a positive relationship with the severity of clinical manifestations [7]. In addition, the norepinephrine elimination mechanism is impaired among POTS patients with significant sympathetic activation [28, 29]. Therefore, attention has been given to the role of hyperadrenaline status in developing POTS. Metoprolol is a beta-adrenoceptor blocker that inhibits sympathetic nerve function [6]. However, due to the complex pathogenesis of POTS, empirical and nonselective use of metoprolol in pediatric POTS usually shows poor effectiveness [5]. Therefore, if the sympathetic hyperactivity or plasma hyperadrenaline status as the primary pathogenesis in patients with POTS can be effectively predicted before treatment, individualized, targeted treatment with β-adrenoceptor blockers would vastly improve the effectiveness of POTS. Many scholars have conducted relevant studies in the past, but they have their own limitations in terms of price, operability, index stability, and so on. It is indispensable to find convenient, easily accessible, non-invasive, simple, and affordable indicators to efficiently predict high sympathetic activity in pediatric POTS before treatment, so that individualized metoprolol treatment can be implemented among POTS patients.

Electrocardiogram waveforms can reflect the effect of sympathetic nerves on the heart, and the autonomic nervous system primarily affects the depolarization and repolarization processes of the myocardium by secreting neurotransmitters and changing the ion distribution on the myocardial cell membrane surface [30–32]. The P wave is a potential change produced by atrial depolarization. During sympathetic stimulation, the action potential of the atrial muscle cell is shortened; the slope of phase 0 increases; the refractory period is compressed; the automaticity is elevated; the triggering activity is enhanced, and the P wave voltage, P wave maximum time, and P wave dispersion can be significantly increased [12, 33]. Hooper et al. [34] observed a significant and positive increase in P wave duration when rats inhaled allyl isothiocyanate (AITC), an experimental agent exciting the sympathetic nerves. Cheema et al. [12] observed that epinephrine infusion prolonged P wave duration in healthy volunteers. In contrast, atropine-based parasympathetic blockade resulted in a reduced P wave duration with excellent reproducibility among subjects (intraclass correlation coefficient 0.99). The above studies indicated that P wave changes were closely related to autonomic nervous function. In this study, the responders showed longer Pcmax, increased Pd, increased P wave amplitude, and shorter Pcmin before metoprolol treatment than the non-responders, indicating that the responders to metoprolol in children with POTS were more likely to have high sympathetic activity before treatment than the non-responders. Therefore, individualized metoprolol treatment would show a better effect on those cases.

QT interval is the total duration of depolarization and repolarization of the myocardial cells, equivalent to the end of phase 0 to phase 3 of the action potential and is mainly formed by changing the ion transport across the membrane, which in turn affects the cardiac electrical activity and is modulated by an autonomic nervous system. Sympathetic stimulation causes elevated heart rate, shortened QT interval, and increased QTd; after vagus nerve activation, the heart rate decreases, while the QT interval is prolonged [13, 35]. According to Huang et al. [36], sympathetic denervation in a canine model significantly decreased HR and prolonged QT duration. Kittnar et al. [37] found that patients with diabetes and cardiac autonomic neuropathy were likely to have tachycardia, QRS, and QT interval shortening, often due to elevated sympathetic tone. Previous studies have shown significant changes within the QTc interval over 24 hours in healthy adults, with subjects awake with shorter QTc than at night [38], consistent with the circadian profile of circulating catecholamine concentrations [39]. The above studies depict that the QT interval is closely related to autonomic function. In this study, metoprolol responders had increased QTd and shorter QTcmin before treatment than non-responders, indicating that such indices would greatly value the individualized metoprolol treatment strategy.

The Tpe interval refers to the duration between the T wave apex and end in ECG, represents the time from the submembranous myocardial repolarization end to the medial M cell repolarization end within the center during cardiac repolarization, and reflects the ECG index of the transmural ventricular repolarization dispersion. Tpe dispersion tends to reflect the spatial discordance of transmural dispersion due to inconsistent repolarization of the three layers of the ventricular wall at different sites. Yagishita et al. [40] reported that the stellate ganglia and the heart were exposed in Yorkshire pigs. Sympathetic nerve stimulation was performed unilaterally or bilaterally, and a significant prolongation of the Tpe interval was observed. Tanabe et al. [41] observed surface electrocardiogram changes before and after epinephrine infusion in 13 cases depicting long QT syndrome (LQTS) type 1 (LQT1) and 6 showing LQTS type 2 (LQT2), and found that epinephrine significantly enhanced the mean Tpe interval and dispersion during LQT1 and LQT2. The increased mean Tpe interval and dispersion caused by epinephrine were more significant among LQT1 cases than LQT2 cases, which could explain the greater sensitive clinical characteristics of LQT1 patients during sympathetic stimulation. Studies indicate that the Tpe interval and its changes are closely associated with high catecholamine levels and sympathetic nerve activity. In this study, Tpemax and Tped in responders were longer before metoprolol treatment than in non-responders; Tpemin was shorter than that in non-responders, and Tpe interval dispersion was enhanced, indicating that responders to metoprolol had high sympathetic activity before treatment. At the same time, metoprolol treatment was more effective in these patients, further verifying the hypothesis that baseline Tpe dispersion could be used as an early predictive indicator of the effectiveness of metoprolol treatment in POTS.

Therefore, P wave duration and amplitude, QT interval, and Tpe dispersion could reflect autonomic function. When sympathetic nerves are excited, heart rate increases; P wave amplitude increases; P wave duration is prolonged, and QT interval shortens. Simultaneously, T waves show inversion or flatness; the Tpe interval is prolonged, and the dispersion increases [12–15]. Based on the clinical significance of the ECG indicators and the results of multicollinearity analysis, pre-treatment Pcmax, QTcmin, Tped from ECG, and demographic characteristics (gender, age, and BMI) were examined through binary logistic regression using the backward stepwise method. A nomogram model to predict the effect of metoprolol treatment in pediatric and adolescent POTS was constructed according to pre-treatment ECG indicators. The model depicted that pre-treatment Pcmax, QTcmin, and Tped showed a better C-index level in the prediction and presented a better correlation with the actual occurrence. The internal validation and external validation also revealed that the nomogram model could effectively predict the effective rate and depicted a better clinical application value.

The present work provided the first nomogram model to predict the effectiveness of metoprolol in pediatric POTS. This predictive model depicted good accuracy and consistency in both samples built into the model and samples validated externally. The measurement of ECG indicators has advantages, such as noninvasiveness, easy operation, and good cost-effectiveness. Meanwhile, the nomogram, a simple visualized graph, enables its application in clinical practice to be more convenient [42]. The above model would provide clinicians with a personalized metoprolol treatment strategy for children and adolescents with POTS.

However, there were some limitations to the study. This was a retrospective and single-center-based study. Therefore, prospectively randomized and controlled studies are needed in the future to assist in predicting early optimization in children with POTS.

In conclusion, in the present pilot study, for the first time, we developed a high-precision nomogram model to assist clinicians during the early decision of metoprolol therapy for children and adolescents suffering from POTS, which is significant in improving the therapeutic ability of POTS among children and adolescents.

## Supplementary Information

Below is the link to the electronic supplementary material.

Supplementary file1 (DOC 67 kb)

## Data Availability

The datasets generated during and/or analyzed during the current study are available from the corresponding author on reasonable request.
