# Supplementary material for: A predictive model of response to metoprolol in children and adolescents with postural tachycardia syndrome
Source: World J Pediatr. 2023 Feb 13;19(4):390–400. doi: 10.1007/s12519-022-00677-4 (PMC10060270; doi:10.1007/s12519-022-00677-4)
Supplement: Supplementary file 1 — Supplementary file1 (DOC 67 kb) [file 12519_2022_677_MOESM1_ESM.doc]

Supplemental table 1 Multi-collinearity analysis of ECG parameters in children with POTS in the training set

| Variables, r and P values | | **P wave amplitude** | **Pcmax** | **Pcmin** | **Pd** | **Pcd** | **QTcmin** | **QTd** | **QTcd** | **Tpemax** | **Tpemin** | **Tped** |
| --- | --- | --- | --- | --- | --- | --- | --- | --- | --- | --- | --- | --- |
| **P wave amplitude** | **r** | 1 | 0.204 | 0.079 | 0.055 | 0.142 | −0.206 | 0.145 | 0.217 | 0.147 | −0.058 | 0.171 |
|  | **P value** | - | 0.047 | 0.447 | 0.599 | 0.170 | 0.045 | 0.162 | 0.034 | 0.155 | 0.575 | 0.097 |
| **Pcmax** | ***r*** | 0.204 | 1 | 0.348 | 0.590 | 0.723 | 0.018 | 0.128 | 0.217 | 0.059 | −0.132 | 0.130 |
|  | **P value** | 0.047 | - | 0.001 | < 0.01 | < 0.01 | 0.860 | 0.215 | 0.034 | 0.570 | 0.203 | 0.211 |
| **Pcmin** | **r** | 0.079 | 0.348 | 1 | −0.522 | −0.396 | 0.316 | −0.232 | −0.141 | −0.080 | 0.263 | −0.223 |
|  | **P value** | 0.447 | 0.001 | - | < 0.01 | < 0.01 | 0.002 | 0.024 | 0.172 | 0.442 | 0.010 | 0.030 |
| **Pd** | **r** | 0.055 | 0.590 | −0.522 | 1 | 0.963 | −0.253 | 0.323 | 0.290 | 0.141 | −0.291 | 0.296 |
|  | **P value** | 0.599 | < 0.01 | < 0.01 | - | < 0.01 | 0.014 | 0.001 | 0.004 | 0.172 | 0.004 | 0.004 |
| **Pcd** | **r** | 0.142 | 0.723 | −0.396 | 0.963 | 1 | −0.215 | 0.296 | 0.317 | 0.117 | −0.323 | 0.291 |
|  | **P value** | 0.170 | < 0.01 | < 0.01 | < 0.01 | - | 0.036 | 0.004 | 0.002 | 0.261 | 0.001 | 0.004 |
| **QTcmin** | **r** | −0.206 | 0.018 | 0.316 | −0.253 | −0.215 | 1 | −0.609 | −0.584 | 0.064 | 0.322 | −0.121 |
|  | **P value** | 0.045 | 0.860 | 0.002 | 0.014 | 0.036 | - | < 0.01 | < 0.01 | 0.538 | 0.001 | 0.245 |
| **QTd** | **r** | 0.145 | 0.128 | −0.232 | 0.323 | 0.296 | −0.609 | 1 | 0.978 | 0.346 | −0.170 | 0.422 |
|  | **P value** | 0.162 | 0.215 | 0.024 | 0.001 | 0.004 | < 0.01 | - | < 0.01 | 0.001 | 0.100 | < 0.01 |
| **QTcd** | **r** | 0.217 | 0.217 | −0.141 | 0.290 | 0.317 | −0.584 | 0.978 | 1 | 0.322 | −0.198 | 0.414 |
|  | **P value** | 0.034 | 0.034 | 0.172 | 0.004 | 0.002 | < 0.01 | < 0.01 | - | 0.001 | 0.055 | < 0.01 |
| **Tpemax** | **r** | 0.147 | 0.059 | −0.080 | 0.141 | 0.117 | 0.064 | 0.346 | 0.322 | 1 | 0.191 | 0.834 |
|  | **P value** | 0.155 | 0.570 | 0.442 | 0.172 | 0.261 | 0.538 | 0.001 | 0.001 | - | 0.064 | <0.01 |
| **Tpemin** | **r** | −0.058 | −0.132 | 0.263 | −0.291 | −0.323 | 0.322 | −0.170 | −0.198 | 0.191 | 1 | −0.382 |
|  | **P value** | 0.575 | 0.203 | 0.010 | 0.004 | 0.001 | 0.001 | 0.100 | 0.055 | 0.064 | - | < 0.01 |
| **Tped** | **r** | 0.171 | 0.130 | −0.223 | 0.296 | 0.291 | −0.121 | 0.422 | 0.414 | 0.834 | −0.382 | 1 |
|  | **P value** | 0.097 | 0.211 | 0.030 | 0.004 | 0.004 | 0.245 | < 0.01 | < 0.01 | < 0.01 | < 0.01 | - |

*ECG* electrocardiogram, *POTS* postural tachycardia syndrome, *Pcmax*, the maximum value of P wave duration in 12 leads of ECG after correction, *Pcmin* the minimum value of P wave duration in 12 leads of ECG after correction, *Pd* P wave duration dispersion, *Pcd* Pd after correction, *QTcmax* the maximum value of QT interval in 12 leads of ECG after correction, *QTcmin* the minimum value of QT interval in 12 leads of ECG after correction, *QTd* QT interval dispersion *QTcd* QTd after correction, *Tpemax* the maximum value of T-peak-to-T-end interval in 12 leads of ECG, *Tpemin* the minimum value of T-peak-to-T-end interval in 12 leads of ECG, *Tped* T-peak-to-T-end dispersion, - not available

Supplemental table 2 Coefficients of binary logistic regression for predicting metoprolol efficacy in children with POTS in the training set

| **Variables** | **B** | **SE** | **Wald** | ***P* value** | **OR** | **95% CI for OR** |
| --- | --- | --- | --- | --- | --- | --- |
| **Pcmax** | 0.073 | 0.032 | 5.314 | 0.021 | 1.076 | 1.011–1.145 |
| **QTcmin** | −0.040 | 0.016 | 5.805 | 0.016 | 0.961 | 0.931–0.993 |
| **Tped** | 0.208 | 0.056 | 13.842 | < 0.01 | 1.231 | 1.104–1.374 |
| **Constant** | −2.258 | 6.395 | 0.125 | 0.724 | 0.105 | - |

*POTS* postural tachycardia syndrome, *ECG* electrocardiogram, *Pcmax*, the maximum value of P wave duration in 12 leads of ECG after correction, *QTcmin* the minimum value of QT interval in 12 leads of ECG after correction, *Tped* T-peak-to-T-end dispersion, - not available, *SE* standard error, *OR* Odds Ratio, *CI* confidence interval
